# Silent dissemination of HTLV-1 in an endemic area of Argentina. Epidemiological and molecular evidence of intrafamilial transmission

**DOI:** 10.1371/journal.pone.0174920

**Published:** 2017-04-06

**Authors:** María C. Frutos, Rene Gastaldello, Marcos Balangero, Carlos Remondegui, Sebastián Blanco, Koko Otsuki, Ana Carolina Paulo Vicente, David Elías, Arnaldo Mangeaud, Silvia Nates, Sandra Gallego

**Affiliations:** 1 Instituto de Virología “Dr. J. M. Vanella”, Facultad de Ciencias Médicas–Universidad Nacional de Córdoba, Córdoba, Argentina; 2 Departamento de Enfermedades Infecciones, Hospital San Roque, San Salvador de Jujuy, Argentina; 3 Laboratorio de Genética Molecular de Microorganismos, Fundação Oswaldo Cruz, Rio de Janeiro, Brazil; 4 Departamento de Matemática. Facultad de Ciencias Exactas, Físicas y Naturales- Universidad Nacional de Córdoba, Córdoba, Argentina; University of Minnesota College of Veterinary Medicine, UNITED STATES

## Abstract

**Background:**

Molecular and epidemiological studies of transmission routes and risk factors for infection by HTLV-1 are extremely important in order to implement control measures, especially because of the high prevalence of HTLV-1 in several regions of the world. San Salvador de Jujuy, Northwest Argentina, is a highly endemic area for HTLV-1 and foci of tropical spastic paraparesis/HTLV-1-associated myelopathy.

**Objective:**

To gain further insight into the role of intrafamilial transmission of HTLV-1 in a highly endemic region in Argentina.

**Method:**

Cross-sectional study in Northwest Argentina. Epidemiological data and blood samples were collected from 28 HTLV-1 infected subjects (index cases) and 92 close relatives/cohabitants. HTLV-1 infection was diagnosed by detection of antibodies and proviral DNA. The LTR region was sequenced and analyzed for genetic distances (VESPA software), in addition to determination and identification of polymorphisms to define HTLV-1 family signatures.

**Results:**

Fifty seven of the 120 subjects enrolled had antibodies against HTLV-1 and were typified as HTLV-1 by PCR. The prevalence rate of HTLV-1 infection in family members of infected index cases was 31.52% (29/92). The infection was significantly associated with gender, age and prolonged lactation. Identity of LTR sequences and presence of polymorphisms revealed high prevalence of mother-to-child and interspousal transmission of HTLV-1 among these families.

**Conclusion:**

There is an ongoing and silent transmission of HTLV-1 through vertical and sexual routes within family clusters in Northwest Argentina. This evidence highlights that HTLV-1 infection should be considered as a matter of public health in Argentina, in order to introduce preventive measures as prenatal screening and breastfeeding control.

## Introduction

Human T-cell Lymphotropic virus type 1 (HTLV-1) has been associated with lymphoproliferative disorders, inflammatory and degenerative diseases of the central nervous system, and some disorders of the immune system [1, 2]. HTLV-1 is the etiological agent of adult T-cell leukaemia (ATL) and tropical spastic paraparesis/HTLV-1 associated myelopathy (TSP/HAM). Cases of HTLV-1 infection and HTLV-1 associated diseases have been reported in almost all South American countries including Brazil, Colombia, Argentina, Peru, French Guyana, and Chile [3]. Moreover, some areas of South America such as the Northeast region of Brazil and Northwest Argentina are considered endemic for HTLV-1 [4, 5].

Infection is considered lifelong and most of the infected subjects remain asymptomatic, becoming viral reservoirs and continuing the chain of transmission [5]. The efficiency of HTLV-1 dissemination is related to the route of transmission [6]. While the main risk of infection in developed countries appears to be the use of contaminated needles by drug users, in developing countries the transmission is mainly intrafamilial [5, 7, 8, 9].

Familial clustering has been studied in Japan, Caribbean and Brazil [2, 9,10,11]. In these areas, vertical transmission remains the most important route of transmission, since it occurs in up to 25% of the children breastfed by seropositive mothers [8, 9].

In 2004, we reported diverse prevalence rates of HTLV-1 infection according to different geographic areas, through an overview of the seroepidemiology of HTLV-1 in Argentina. In fact, the endemic zone in the North area of the country has a prevalence of 0.6–1.2% in blood donors while in non-endemic parts (e.g., central region of the country), the prevalence in blood donors is <0.1% [4]. A second study conducted in 2008 in Jujuy province (Northwest area of Argentina) allowed us to identify HTLV-1aA genetic subtype from a large set of HTLV-1 isolates (n = 65) from descendants of Amerindians who live in this region and HTLV-1aB genetic subtype of Japanese subgroup in descendants of non-Japanese people in Argentina [12].

There are few published data about family transmission of HTLV-1 in Argentina based on epidemiological and molecular evidence. While we have shown that HTLV-1 is familiarly transmitted in a non-endemic area of Argentina [13], reliable data on the transmission of HTLV-1 in endemic areas of the country is still limited. Thereby, studies regarding HTLV-1 transmission routes are extremely important, especially given the prevalence of HTLV-1 in several regions of the country [13–16]. Therefore, to gain further insight into the familial transmission of HTLV-1 in Argentina, we focused our research on 28 family groups randomly selected from several family clusters belonging to HTLV-1-infected subjects living in the province of Jujuy, an endemic area in the Northwest part of the country.

## Materials and methods

In a cross-sectional study, epidemiological data and blood samples were collected from 28 HTLV-1 infected subjects (index cases) randomly selected from the records of San Roque Hospital, San Salvador de Jujuy and 92 members of their family groups. Samples were codified as J-family number-order of sampling. HTLV-1 infection in these 28 index cases had been diagnosed by detection of antibodies against HTLV-1 and amplification of proviral DNA. The 92 family members were included in the study based on the criteria of being a close relative of an index case or cohabiting in the same household.

This study complied with the principles outlined by the Declaration of Helsinki and was approved by the Ethics Committee of San Roque Hospital, San Salvador de Jujuy, Argentina (CIES 22/04, 2015). Written informed consent was signed by all the participants or their parents/legal guardians prior to sample collection. HTLV-1 infected subjects were counseled by a multidisciplinary team that offered information about the infection and medical support.

At enrolment, a structured questionnaire was performed by trained interviewers to collect epidemiological and social data, parenteral exposure and sexual history. Each subject was tested for HTLV-1 and HTLV-2 antibodies by ELISA 3.0 (MP Diagnostic, Santa Ana, CA) and Particle Agglutination (PA) assay (Serodia Fujirebio Inc. Tokyo, Japan). Reactive samples were further tested with an ‘‘in house” indirect immunofluorescence assay (IFA) on MT-2 and Mo-T cell lines [17] and Western blot 2.4 (MP Diagnostic, Santa Ana, CA) according to the manufacturer’s instructions. All serum samples reactive by screening and positive by IFA and Wb were considered definitely positive for HTLV-1 or HTLV-2 infection.

High molecular weight DNA was extracted from blood samples of all 120 subjects. Polymerase chain reaction (PCR) was carried out to amplify 219-bp of the *tax* gene following the protocols described by Vandamme et al. 1997 [18]. All HTLV-1/2 samples that were positive by generic PCR were subsequently typed by specific nested-PCR for HTLV-1 (100 bp) and HTLV-2 (151 bp), targeting the *tax* region [18]. PCR products were separated on 2% agarose gel and visualized under UV light after ethidium bromide staining.

Amplification of 672 bp of the LTR region of the viral genome was carried out in all positive samples, as previously described [19]. PCR products corresponding to HTLV-1 LTR sequence were purified using QIAamp PCR purification kit (Qiagen, Hilden, Germany) according to the manufacturer’s instructions. The LTR fragments were directly sequenced on both strands from the internal primer set using Big Dye Terminator Cycle Sequencing Ready reaction Kit and ABI 377 automated DNA sequencer (Applied Biosystems). The alignment of 552 nucleotides of LTR from isolates of family members was performed using Clustal W and compared with Pairwise/Blast/NCBI. LTR sequences were subjected to signature pattern analysis (Genetic distances) with VESPA software [20] and the presence of common family polymorphisms was visually confirmed. Sequences from this study were deposited in GenBank (Accession number DQ182355-DQ182415).

Frequency distribution of each variable was evaluated by Chi-square, Fisher’s exact test or Contingency Coefficient and Cox-Mantel-Haenszel tests.

A bivariate analysis was also carried out. Odds ratios (ORs) and exact 95% confidence intervals (CIs) were calculated to determine the association between selected variables and HTLV-1 infection. In addition, test of difference between proportions was applied. The statistical package R version 3.2.4 software (R Core Team) was used for statistical analyses. P-values <0.05 were considered significant.

## Results

Fifty-seven of the 120 subjects enrolled in this study (including the 28 HTLV-1 index cases) had antibodies against HTLV-1. All these cases were confirmed and typified as HTLV-1. The prevalence of HTLV-1 infection in close relatives/cohabitants of the infected index cases was 31.52% (29/92).

The overall genetic distance observed on HTLV-1 LTR (552bp) considering all infected family members ranged from 0.0 to 1.4%.

**Fig 1:**
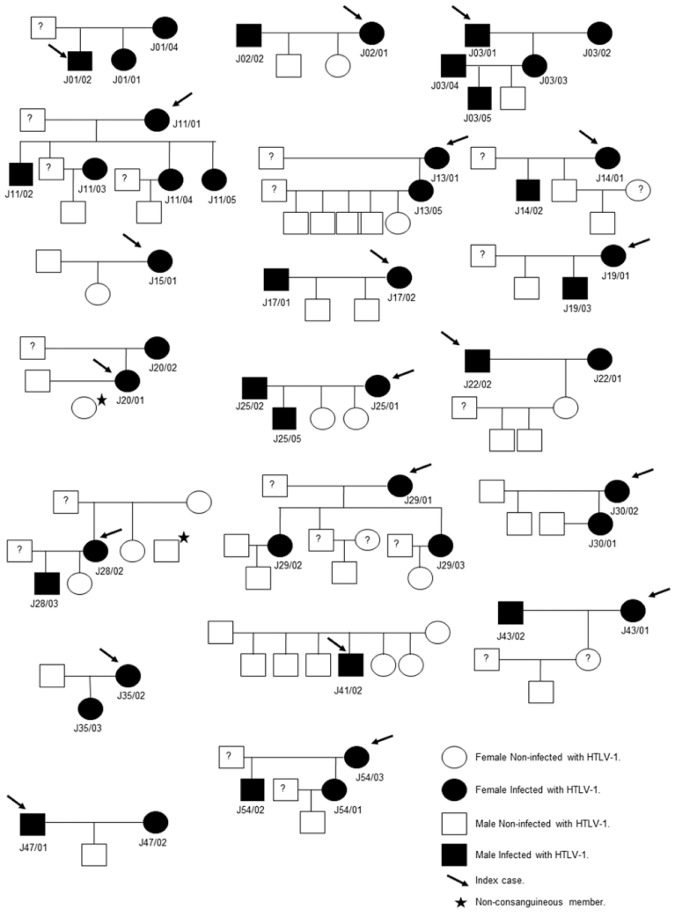
Family tree of index cases infected with HTLV-1.

Except for two families (J54 and J11), genetic distance between viral isolates of each family group was 0.0%, showing 100% identity. Epidemiological data, along with molecular results evidenced that familial transmission of HTLV-1 occurred in 19 of the 28 (67.9%) family groups. In all these families, at least two members were infected with HTLV-1 (Fig 1).

Isolates from members of Family J01 showed 100% identity. Furthermore, a common family polymorphism at position 746 on LTR respect to ATK reference strain was found (Table 1). In this family, both the son (J01/02-index case) and daughter (J01/01) were breastfed by their HTLV-1 positive mother (J01/04) for more than 1 year; thus, vertical transmission was demonstrated.

**Table 1:** Common familiar polymorphisms on 552 nucleotides of LTR sequence from family isolates of Jujuy, considering J02029 as reference strain.

Family	Polymorphism
J01	746 T/C
J03	561 A/C
J11*	212 C/G
J14	711 G/A
J15**	711 G/A
J16	632 C/T
J17	441 C/T
J20	212 C/T; 646 A/G
J22	233 A/G; 628 T/C
J43	663–682 Insertion of 20 nucleotides.

* All members, except J11/03 had the indicated polymorphism.

** J15-01 is daughter of index case J14/01.

Sexual transmission was demonstrated in Families J02 and J47 showing 100% sequence identity between spouses.

The isolates from Family J03 were 100% identical. Furthermore, a common family polymorphism at position 561 on LTR respect to ATK reference strain was observed (Table 1). In this family, vertical and sexual transmission was demonstrated.

The isolates from the infected brothers of Family J11 (J11/02, J11/04, J11/05) showed 100% sequence identity between them and with their HTLV-1 positive mother (J11/01, index case); all of them had a common polymorphism at position 212 on LTR respect to ATK reference strain (Table 1). Despite the fact that intrafamilial transmission of the virus was demonstrated in most of the members in this family, subject J11/03 probably acquired the infection outside the intimate circle, since he showed 0.4% genetic distance respect to the other members of the family as well as absence of polymorphism at position 212 on LTR.

Genetic distance was 0.0% between J13/01 (HTLV-1 infected mother) and J13/05, demonstrating vertical transmission; moreover, the epidemiological data showed that daughter J13/05 had been breastfed for more than 1 year.

Mother (J14/01-index case) and son (J14/02) of Family J14 presented 100% viral sequence identity, demonstrating vertical transmission. Moreover, a common family polymorphism at position 711 on LTR respect to ATK reference strain was found (Table 1). The son J14/02 had been breastfed for 1 year.

In Family J16, isolates from mother (J16/01-index case) and son (J16/03) had 100% viral sequence identity, indicating vertical transmission. Moreover, a common family polymorphism at position 632 on LTR respect to ATK was identified (Table 1). Although it was not possible to study her husband, the epidemiological history of J16/01 referred that she had worked as a prostitute and had history of other sexually transmitted disease such as syphilis and gonorrhea.

The isolates from spouses of Family J17 had 100% sequence identity, showing a family polymorphism at position 441 on LTR respect to ATK (Table 1). Thus, sexual transmission of the infection was demonstrated.

Vertical transmission was demonstrated also in the mother of Family J19 (J19/01-index case) and her son (J19/03). They had 100% sequence identity and the epidemiological data showed that the child had been breastfed for 2 years.

In Family J20, LTR sequence identity of 100% was found between mother (J20/02) and daughter (J20/01-index case), who had been breastfed for more than 1 year. Moreover, common polymorphisms at positions 212 and 646 on LTR respect to ATK were detected (Table 1).

In Family J22, sexual transmission between spouses J22/01 and J22/02 was observed (0.0% genetic distance) and two common polymorphisms at positions 233 and 628 on LTR respect to ATK were found (Table 1). Both spouses had history of blood transfusions.

In Family J25, vertical and sexual transmission of the virus (100% sequence identity) was demonstrated. Epidemiological data showed that the infected son (J25/05) had been breastfed by his mother (J25/01) for more than 2 years.

Vertical transmission was demonstrated from mother (J28/02-index case) to son (J28/03) in Family J28 (100% sequence identity); the son had been breastfed during 2 years.

In Family J29, vertical transmission was established since the daughters (J29/02 and J29/03) had 100% sequence identity compared to their mother (J29/01); they were breastfed for more the 1 year.

In Family J30, there was 100% identity between virus from mother (J30/02-index case) and daughter (J30/01), demonstrating vertical transmission. The same route of transmission was observed in Family J35 between mother (J35/02-index case) and daughter (J35/03), also with 100% of LTR sequence identity.

Sexual transmission was demonstrated in Family J43, with 100% sequence identity between spouses (J43/01 and J43/02). Moreover, an insertion of 20 nucleotides at positions 663–682 on LTR was exclusively observed in both isolates (Table 1).

Higher genetic distance was noticed between isolates of Family J54, in which virus sequence from mother (J54/03) and daughter (J54/01) were 100% identical and the genetic distance between the virus from mother (index case) and son (J54/02) was 1.4%. Thus, the son J54/02 would have acquired the infection outside the family (Fig 1).

From the 9 family groups that had only one infected subject (data not shown), familial transmission was demonstrated only for the index case of Family J15 (J15/01) and individual transmission for Family J41 (J41/02).

Index case J15/01 was daughter of the index case of Family J14 (J14/01) (Fig 1). There was 100% sequence identity between their viruses and J15/01 also had the polymorphism common for Family J14 at position 711 on LTR respect to ATK reference strain (Table 1). This issue, along with the fact that she had been breastfed for more than 5 years, demonstrated vertical transmission of the virus from mother to daughter.

On the other hand, individual transmission of HTLV-1 was demonstrated in one of the subjects of Family J41 by the absence of HTLV-1 infection in all family members (parents and brothers of J41/02). J41/02 probably acquired infection through the sexual route, since his partner had history of intravenous drug usage (Fig 1). No data about HTLV-1 conditions were available for his sexual couple.

Vertical and sexual transmission occurred in 14 (73.7%) and 7 (36.8%) of the 19 families, respectively. Vertical transmission was confirmed in 20 (40%) of the 50 mother/children pairs, while sexual transmission occurred in 8 (50%) of the 16 couples. Thereby, there were not significant differences between sexual and vertical routes for HTLV-1 transmission in this population (95% CI: 0.0034 to 0.5299 p = 0.057). Moreover, no significant differences were found in the frequency of transmission from mothers to daughters (50%, 11/22) compared to mothers to sons (32.1%, 9/28) (95% CI: 0.1333 to 0.4904 P = 0.3228) (Table 2).

**Table 2:** Frequency of HTLV-1 infection in mother-offspring and spouse-spouse pairs in the endemic area of Argentina.

Route of transmission	Number of subjects studied	Number of subjects infected with HTLV-1		
		N (%)	95% CI	P-value
**Vertical**			0.1333 to 0.4904	0.3228
daughters	22	11 (50)		
sons	28	9 (32.1)		
**Sexual**			0.2158 to 0.673	0.4642
wives	10	8 (80)		
husbands	14	8 (57.1)		

Most of the infected subjects were females (37/57; 65.0%), mean age: 36.3 years (SD±22.1), ranging from 3 to 83 years; the majority of them (49.1%) were aged between 30 and 60 years.

The bivariate analysis showed that HTLV-1 infection was significantly associated with female sex, age over 18 years, and length of lactation (Table 3). Prolonged breastfeeding from a HTLV-1 positive mother constituted a risk factor for HTLV-1 infection. Its extension for more than 1 year was observed in most (70%) of the seropositive relatives and less than 1 year in 57.14% of seronegative ones (95% CI: 1.49 to 6.63 *p* = 0.0026) (Table 3).

**Table 3:** Variables associated with HTLV-1 infection among families in the endemic area of Argentina.

Bivariate analysis					
Variable	HTLV-1 negative	HTLV-1 positive	Odds ratio	95% CI	*p-*value*
	N (%)	N (%)			
**Gender**					
M	38 (65.5)	20 (34.5)	2.81	1.34–5.86	0.0057
F	25 (40.3)	37 (59.6)			
**Age (years)**					
≤ 18	33 (89.2)	4 (10.8)	14.58	4.95–42.91	<0.0001
> 18	30 (36.1)	53 (63.9)			
**Length of lactation (years)**					
≤1	36 (67.9)	17 (32.1)	3.14	1.49–6.63	0.0026
>1	27 (40.3)	40 (59.7)			

*p value for chi-squared test.

Despite the fact that prevalence of HTLV-1 in wives (80%, 8/10) was higher than in husbands (57.1%, 8/14), this difference was not significant (95% CI: 0.2158 to 0.673 P = 0.4642) (Table 2). All the couples declared a stable relationship with an average length of 13.9 years in cases of seronegative spouses and 28.3 years in seropositive ones.

The HTLV-1 genetic analysis determined that all sequences belonged to Cosmopolitan subtype HTLV-1a Transcontinental subgroup A, in Latin American cluster (data not show). These results are in agreement with data previously reported by our group [12].

## Discussion

In this study, a systematic research concerning familiar transmission of HTLV-1 infection in an endemic area of Northwest Argentina was performed for the first time. We evaluated 28 HTLV-1 infected subjects (index cases) selected from the records of San Roque Hospital, San Salvador de Jujuy, in addition to 92 members of their family groups.

As HTLV-1 infection had been suspected to spread silently within families in North Argentina based on the observations of physicians about the usual outcome of TSP in family groups, we aimed to investigate the aggregation of this infection among close relatives/cohabitants of the index cases. Previous studies from Colombia, Chile and Brazil have shown high rates of HTLV-1 infection in relatives of TSP/HAM patients [7, 21–23].

The results presented herein reflect a high prevalence of HTLV-1 infection (31.5%) in people cohabiting with the index cases, pointing out the great importance of familial dissemination of HTLV-1 infection in North Argentina. In agreement with what happens in the rest of the world, the highest rate of infection was found in women (Table 3) [2, 24–27].

High prevalence rates of HTLV-1 infection in viral-carrier families have been demonstrated in several regions worldwide. Previous studies in high-endemic areas of Brazil and Africa have reported prevalence rates similar to values found in this study [2, 28], corroborating that Jujuy province is a high endemic area for HTLV-1 infection, with prevalence rates much higher than values recognized in other regions of the country [29–31]. In other high-endemic areas of the world, such as Japan, the prevalence of HTLV-1 is 38.5% among relatives of infected individuals [32]. Moreover, Lu et al [33] have described similar prevalence rates in 20 family members of HTLV-1-seropositive blood donors in Taiwan.

The seroprevalence rate for HTLV-1 described herein for family members of HTLV-1 carriers is markedly higher than the mean rate of infection of blood donors in Jujuy (1.0%) [4]. This finding reinforces the need to investigate the different transmission routes involved to gain insight into the epidemiology of HTLV-1 in this region, which is helpful in decision-making and scheduling preventive measures.

Regarding the routes of transmission, during years it was suspected that in Jujuy, HTLV-1 infection was acquired through lactation and sexual routes, similar to other regions in the world, but so far, no systematic studies had been carried out to clarify the importance of these routes for HTLV-1 dissemination in North Argentina. Herein, we present epidemiological and molecular evidence of this fact, evidencing high rates of intrafamilial transmission of HTLV-1, since transmission of the virus was demonstrated inside 19 of the 28 (67.9%) families studied (Fig 1).

High levels of vertical (73.7%) and sexual (36.8%) transmission were demonstrated for family groups with more than one infected subject. Rates of HTLV-1 infection found in mother-child pairs (40.0%) were higher than values reported in Brazil, where the overall prevalence of HTLV infection in mother-child pairs is 20.4% [2].

We found a high rate (70%) of HTLV-1 infection among children breastfed for more than 1 year (Table 3). Moreover, we demonstrated, through epidemiological and molecular data, that vertical transmission occurred in 37.5% of them (15/40). This rate is similar to values described in our neighbor country Brazil for children breastfed for more than 6 months (34.0%) [2]. The implication of prolonged lactation was also demonstrated in Peru in an investigation of 120 HTLV-1-infected mothers. In that study, the overall prevalence of infection was 23.3% (28/120) among children breastfed less than six months and 32.6% (23/76) in those breastfed for more than 6 months [34].

Several studies have suggested that vertical transmission is more prevalent in women than in men [7, 35]. Nevertheless, in a recent study carried out in a Brazilian cohort, no differences between sons and daughters were demonstrated [2]. In agreement with that study, the infection rates in our population were similar between sons and daughters (Table 2).

Our results clearly corroborated that infection by HTLV-1 was mainly concentrated among women (Table 3) and despite there was not a significant difference (95% CI: 0.918 to 10.743, p = 0.0937), we observed that the rate of HTLV-1 infection was threefold higher in women than in men older than 18 years old. This is probably related with the sexual acquisition of the infection, as previously proposed [36]. Thus, while we did not find any differences between infections in wives and husbands (Table 2), our results suggest higher risk of sexual transmission from men to women. As a consequence, the infection tends to become more prevalent in female members of the families over time (Table 3).

Even though there were not significant differences between vertical and sexual transmission of HTLV-1 in our population and both routes proved to be relevant, we consider that the importance of sexual transmission might be underestimated. This may be due in part to the limitation of our study to include all sexual partners (husbands). These subjects could not be studied because it is a cultural trait that men of this population live away from their homes, working in diverse temporary jobs.

Molecular analysis demonstrated that HTLV-1 isolates from this region of Argentina are highly preserved (Table 1). Nucleotide analysis of these isolates was useful to confirm the route of HTLV-1 transmission. Therefore, the identity of the LTR sequences in association with epidemiological data confirmed intrafamilial transmission involving horizontal and vertical events, as previously shown. The same LTR sequence had been previously used to establish HTLV transmission routes in other populations [9, 13].

Molecular and epidemiological analysis showed that most of the infected subjects, except for two of them, acquired the infection from a close relative (Fig 1, Table 1). Overall, the results of this study are generally in agreement with previously published data [2, 8, 9, 13, 36, 37] and provide evidence that HTLV-1 is transmitted ‘‘silently” within family clusters in Argentina.

Although prevention of viral spread from the clusters depends on the identification of cases in the population, in Jujuy, as in overall Argentina, infection by HTLV is not currently considered as a public health problem and has been largely neglected. Regarding this fact, we agree with Costa et al. when they say that “HTLV is an example of the key neglected infectious diseases, which have been identified as priorities for research in Latin America and the Caribbean” and “Overall, the general ignorance of the virus increases the risk of transmission considerably” [2].

Since year 2000, we have been carrying out a systematic investigation of HTLV infection in families of virus carriers detected in blood banks in the province of Córdoba (main inland province of Argentina). We demonstrated that intrafamily transmission of HTLV-1 also occurs in this non-endemic area of the country [13] and thanks to the detection of infected families, counseling for prevention of further HTLV transmission has been possible.

Despite North Argentina is recognized as a high endemic area for HTLV, no prevention is being carried out and although HTLV is regularly tested in blood banks, pregnant women are not systematically evaluated. We think, based on the observation of sociodemographic characteristics of the studied population (data not shown) and in agreement with other authors [23, 38, 39], that this is a neglected disease in Argentina because it occurs in the poor, who have little access to the public health system.

The results presented herein demonstrate a strong familial dissemination of HTLV-1 in North Argentina and point out that this infection should be considered a matter of public health in this country.
